# Steps per Day and All-Cause Mortality in Middle-aged Adults in the Coronary Artery Risk Development in Young Adults Study

**DOI:** 10.1001/jamanetworkopen.2021.24516

**Published:** 2021-09-03

**Authors:** Amanda E. Paluch, Kelley Pettee Gabriel, Janet E. Fulton, Cora E. Lewis, Pamela J. Schreiner, Barbara Sternfeld, Stephen Sidney, Juned Siddique, Kara M. Whitaker, Mercedes R. Carnethon

**Affiliations:** 1 Institute for Applied Life Sciences, Department of Kinesiology, University of Massachusetts, Amherst; 2 Department of Epidemiology, University of Alabama at Birmingham; 3 Division of Nutrition, Physical Activity, and Obesity, National Center for Chronic Disease Prevention and Health Promotion, Centers for Disease Control and Prevention, Atlanta, Georgia; 4 Division of Epidemiology and Community Health, University of Minnesota, Minneapolis; 5 Division of Research, Kaiser Permanente Northern California, Oakland; 6 Department of Preventive Medicine, Northwestern University Feinberg School of Medicine, Chicago, Illinois; 7 Department of Health and Human Physiology, Department of Epidemiology, University of Iowa, Iowa City

## Abstract

**Importance:**

Steps per day is a meaningful metric for physical activity promotion in clinical and population settings. To guide promotion strategies of step goals, it is important to understand the association of steps with clinical end points, including mortality.

**Objective:**

To estimate the association of steps per day with premature (age 41-65 years) all-cause mortality among Black and White men and women.

**Design, Setting, and Participants:**

This prospective cohort study was part of the Coronary Artery Risk Development in Young Adults (CARDIA) study. Participants were aged 38 to 50 years and wore an accelerometer from 2005 to 2006. Participants were followed for a mean (SD) of 10.8 (0.9) years. Data were analyzed in 2020 and 2021.

**Exposure:**

Daily steps volume, classified as low (<7000 steps/d), moderate (7000-9999 steps/d), and high (≥10 000 steps/d) and stepping intensity, classified as peak 30-minute stepping rate and time spent at 100 steps/min or more.

**Main Outcomes and Measures:**

All-cause mortality.

**Results:**

A total of 2110 participants from the CARDIA study were included, with a mean (SD) age of 45.2 (3.6) years, 1205 (57.1%) women, 888 (42.1%) Black participants, and a median (interquartile range [IQR]) of 9146 (7307-11 162) steps/d. During 22 845 person years of follow-up, 72 participants (3.4%) died. Using multivariable adjusted Cox proportional hazards models, compared with participants in the low step group, there was significantly lower risk of mortality in the moderate (hazard ratio [HR], 0.28 [95% CI, 0.15-0.54]; risk difference [RD], 53 [95% CI, 27-78] events per 1000 people) and high (HR, 0.45 [95% CI, 0.25-0.81]; RD, 41 [95% CI, 15-68] events per 1000 people) step groups. Compared with the low step group, moderate/high step rate was associated with reduced risk of mortality in Black participants (HR, 0.30 [95% CI, 0.14-0.63]) and in White participants (HR, 0.37 [95% CI, 0.17-0.81]). Similarly, compared with the low step group, moderate/high step rate was associated with reduce risk of mortality in women (HR, 0.28 [95% CI, 0.12-0.63]) and men (HR, 0.42 [95% CI, 0.20-0.88]). There was no significant association between peak 30-minute intensity (lowest vs highest tertile: HR, 0.98 [95% CI, 0.54-1.77]) or time at 100 steps/min or more (lowest vs highest tertile: HR, 1.38 [95% CI, 0.73-2.61]) with risk of mortality.

**Conclusions and Relevance:**

This cohort study found that among Black and White men and women in middle adulthood, participants who took approximately 7000 steps/d or more experienced lower mortality rates compared with participants taking fewer than 7000 steps/d. There was no association of step intensity with mortality.

## Key Points

**Question:**

Are step volume or intensity associated with premature mortality among middle-aged Black and White women and men?

**Findings:**

In this cohort study of 2110 adults with a mean follow-up of 10.8 years, participants taking at least 7000 steps/d, compared with those taking fewer than 7000 steps/d, had a 50% to 70% lower risk of mortality. There was no association of step intensity with mortality regardless of adjustment for step volume.

**Meaning:**

This cohort study found that higher daily step volume was associated with a lower risk of premature all-cause mortality among Black and White middle-aged women and men.

## Teaser

This cohort study assesses the association of step volume and intensity with premature all-cause mortality among Black and White participants in the Coronary Artery Risk Development in Young Adults study.

## Introduction

Regular physical activity is one of the most important behaviors people can do to improve or maintain good health. Being physically active provides substantial health benefits for many conditions, such as cardiovascular disease, diabetes, and several cancers, as well as improving quality of life.^1^ The number of steps people take each day is a meaningful metric for quantifying total daily activity.^2^ The simplicity of the metric and the ease of measurement by wearable devices provides an opportunity for population-wide monitoring of steps. National guidelines for physical activity do not include step counts as a public health target owing to the limited number of studies demonstrating the prospective associations of step volume and intensity with clinical outcomes, including mortality.^1^

Most prospective studies on steps and health include samples of older adults, whereas few studies include adults earlier in their life course or racially diverse populations.^3^ A systematic review by Hall et al^3^ found significant associations between device-measured step volume and all-cause mortality, primarily in older adult populations. The National Health and Nutrition Survey (NHANES) and Norwegian Physical Activity Surveillance Study begin to extend these findings to middle through older adult ages.^4,5^ The Physical Activity Guidelines Advisory Committee identified a need for research in populations with diverse individual characteristics, including age, race, and sex, since these may impact the associations of daily steps and health.^6^ Our study extends research by examining a prospective cohort of middle-aged men and women of Black and White race and the association of steps with premature mortality, considered deaths earlier than US population mean life expectancy.^7^

National guidelines for physical activity recommend at least 150 min/wk of moderate- to vigorous-intensity physical activity, based on scientific evidence supporting intensity of physical activity as important for health benefits.^8^ A 2021 study by Wang et al^9^ of more than 400 000 adults demonstrated the importance of accumulating moderate- to vigorous-intensity physical activity with mortality outcomes. Furthermore, Wang et al^9^ also found greater reductions in mortality for vigorous-intensity activity than for moderate-intensity activity. It remains unclear whether stepping intensity is associated with mortality risk. Some epidemiologic studies have suggested that self-defined walking pace is associated with mortality.^10,11^ Recent studies of steps and mortality have reported that the number (volume) of steps, rather than the intensity (pace or cadence) of steps, was associated with mortality.^4,12^ Studies comparing mortality by step volume and step intensity among middle-aged adults are lacking. These studies are important because intensity requirements to achieve health benefits may differ between older and younger adults.^13,14^

In response to the need for empirical data on the associations of step volume and intensity with mortality in younger and diverse populations, we conducted a prospective study in middle-aged Black and White adults followed up for mortality for approximately 11 years. The objectives of our study were to examine the associations of step volume and intensity with mortality overall and by race and sex.

## Methods

This cohort study received institutional review board approvals from each field center institution. All participants provided written informed consent. This study followed the Strengthening the Reporting of Observational Studies in Epidemiology (STROBE) reporting guideline for cohort studies.

### Study Sample

The Coronary Artery Risk Development in Young Adults (CARDIA) study included 5115 adults aged 18 to 30 years at baseline in 1985 to 1986.^15^ The study recruited a balanced sample by race (Black and White), sex, age, and education from 4 US locations (Birmingham, Alabama; Chicago, Illinois; Minneapolis, Minnesota; and Oakland, California). Participants underwent in-person examinations at baseline and years 2, 5, 7, 10, 15, 20, 25, and 30. Retention rates among surviving participants at examinations were 91% at year 2, 86% at year 5, 81% at year 7, 79% at year 10, 74% at year 15, 72% at year 20, 72% at year 25, and 71% at year 30. At the year 20 follow-up examination (2005-2006; cohort age 38 to 50 years), 2332 participants wore an accelerometer as part of the CARDIA fitness ancillary study.

### Primary Exposure: Step Metrics

Participants were instructed to wear an ActiGraph 7164 accelerometer on the hip, secured by an elastic belt, for 7 consecutive days during all waking hours, removed during sleep and water-based activities.^16^ The ActiGraph 7164 provides valid estimates of free-living steps per day.^17^ Vertical axis data were processed using 60-second epochs, based on a modified SAS program developed for the 2003 to 2004 wave of NHANES.^18,19^ Participants with at least 3 days with at least 10 h/d of wear time were included.^2,20^

Step volume was calculated summing raw step counts directly measured from the accelerometer for each valid day, then calculating the mean across days.^4^ We categorized participants into 3 groups: low (<7000 steps/d), moderate (7000 to <10 000 steps/d), and high (≥10 000 steps/d). The 2011 American College of Sports Medicine position statement suggests adults take at least 7000 steps/d based on norm-referenced pedometer indices.^21^ It has not been determined whether the traditional 10 000 steps/d target is required for health benefits; therefore, we selected this as our high step group to investigate levels of 10 000 steps/d or more. Stepping intensity was based on metrics used in similar epidemiological studies.^4,12^ We calculated the highest steps/min observed in any 30 minutes, not necessarily consecutive, throughout the day, and calculated the mean across all days.^22^ We also quantified intensity as daily minutes at 100 steps/min or more, suggested as moderate intensity.^22^

### Primary Outcome: All-Cause Mortality

The primary outcome of this study was all-cause mortality. Participants or designated proxies were contacted twice yearly to ascertain vital status; searches of the National Death Index were conducted every 5 years.^23^ Staff requested death certificates, hospital records for deaths, and autopsy reports. The CARDIA Endpoints Surveillance and Adjudication Subcommittee used pre-established criteria to classify underlying cause of death. For this analysis, participants were followed up for death through August 31, 2018.

### Covariates

Covariates were assessed at the same examination (2005-2006) as accelerometry. Age, race, and education were queried through self-report during visits. Smoking history classified participants as current, former, or never smokers. Race was included as a covariate in analyses because mortality rates are higher for Black adults compared with White adults. Collection of race data allowed for assessment for potential differential associations of steps with mortality by race. Body weight and height were measured to the nearest 0.5 lbs. and 0.5 cm, respectively, and used to calculate body mass index (BMI; calculated as weight in kilograms divided by height in meters squared). Alcohol intake (mL/d) was collected using an interview administered questionnaire and grouped as 0 mL/d, less than 12 mL/d, or 12 mL/d or more of ethanol (median of the sample reporting any alcohol). Diet was quantified based on the Healthy Eating Index from CARDIA’s food frequency questionnaire.^24^ Participants self-reported their health status, and health status was dichotomized as poor or fair vs good, very good, or excellent. Fasting glucose and total cholesterol were measured using standard laboratory techniques.^15,25,26^ Three blood pressure measurements were taken, and the mean of the last 2 measurements was calculated. Participants self-reported medication use for hypertension, diabetes, and high cholesterol. Current clinical guidelines or reported medication use defined hyperlipidemia, diabetes, and stage 2 hypertension.^27,28,29^ Two physicians adjudicated cardiovascular disease, including coronary heart disease, heart failure, stroke, transient ischemic attack, or peripheral artery disease.

### Statistical Analysis

#### Step Volume

The analytic sample for step volume included 2110 participants with adherent accelerometer wear and covariates collected at year 20 (eFigure 1 and eTable 1 in the Supplement). Cox proportional hazards regression models were used to calculate hazard ratios (HRs) and 95% CIs for the association of step volume groups with all-cause mortality. Model 1 adjusted for age, race, sex, and accelerometer wear time. Model 2 added field center, maximal education attainment through follow-up, smoking, alcohol intake, and BMI. Model 3 added systolic blood pressure, hypertension medications, diabetes, hyperlipidemia, cardiovascular disease, and self-rated health. Less than 5% of data were missing for categorical variables of smoking, alcohol intake, and self-rated health; therefore, missing data were classified as not reported/unknown in our models. Tests for trends across step groups were examined using ordinal values. Restricted cubic splines with knots set at the 10th, 50th, and 90th percentiles of the steps/d distribution examined the dose-response association with mortality.^30,31^ There were no significant interactions between step groups by race and by sex with multiplicative terms. However, because of the paucity of studies in diverse populations, we decided a priori to conduct stratified analyses by race and by sex.

#### Step Intensity

Cox regression analyses using the same modeling strategy were completed for the association of stepping intensity with all-cause mortality. Since there are no established thresholds for stepping intensity, we grouped participants into tertiles. To account for the correlations between step volume and peak 30-minute intensity and time spent at 100 steps/min or more (step volume: *r* = 0.70; 30-minute intensity: *r* = 0.56), we included a model (model 4) with step volume using the residual method. In this method, the intensity variable was regressed on step volume. We added the intensity residual to the model as the independent variable and adjusted for step volume.^12,32^

#### Sensitivity Analyses

We conducted several sensitivity analyses. To examine the potential impact of reverse causation, we removed participants who died within the first 2 years of follow-up. To determine the robustness of covariate adjustments, we performed extended Cox models using time-varying covariates, accounting for covariates during the follow-up period at year 20, 25, and 30 examinations. To determine whether diet influenced the association, we conducted analyses in 1899 participants with dietary data, with and without additional adjustment for the Healthy Eating Index Score. Proportional hazards assumption was not violated when testing statistical significance of interactions between follow-up time and exposures.

Two-sided *P* < .05 was considered statistically significant. Data were analyzed in 2020 to 2021. All analyses were conducted using SAS version 9.4 (SAS Institute) and R statistical software version 4.0 (R Project for Statistical Computing).

## Results

The analytic sample included 2110 participants, with mean (SD) age 45.2 (3.6) years at the year 20 examination and with 1205 (57.1%) women and 888 (42.1%) Black participants. Half of participants took over 9000 steps/d (median [IQR] 9146 [7307-11 162] steps/d). Steps were grouped into low (median [IQR], 5837 [5166-6392] steps/d), moderate (median [IQR], 8502 [7822-9278] steps/d), and high (median [IQR], 11 815 [10 826-13 588] steps/d) step categories (Table 1). There was a significantly greater proportion of women (280 women [62.5%]) and Black participants (241 participants [53.8%]) in the lowest step group (*P* < .001). Participants in the low step group had higher BMI, lower self-rated health, and higher prevalence of stage 2 hypertension and diabetes than the moderate and high step volume groups (Table 1). Mean (SD) time from baseline to end of follow-up was 10.8 (0.9) years. During 22 845 person-years of follow-up, 72 of 2110 participants (3.4%) died. The leading causes of death were cancer (18 participants [25.0%]) and cardiovascular disease (17 participants [23.6%]) (eTable 2 in the Supplement).

**Table 1.** Descriptive Characteristics of Participants at the Year 20 Examination for Total Sample and by Steps Volume Groups

Characteristic	No. (%)			
	Total (N = 2110)	Step group, steps/d		
		Low: <7000 (n = 448)	Moderate: 7000-9999 (n = 863)	High: ≥10 000 (n = 799)
Sex				
Women	1205 (57.1)	280 (62.5)	505 (58.5)	420 (52.6)
Men	905 (42.9)	168 (37.5)	358 (41.5)	379 (47.4)
Age, mean (SD), y	45.2 (3.6)	45.2 (3.8)	45.3 (3.6)	45.2 (3.5)
Race				
Black	888 (42.1)	241 (53.8)	356 (41.3)	291 (36.4)
White	1222 (57.9)	207 (46.2)	507 (58.7)	508 (63.6)
Education, mean (SD), y	15.6 (2.6)	15.3 (2.6)	15.8 (2.5)	15.6 (2.7)
BMI				
Mean (SD)	29.0 (7.0)	32.1 (7.8)	29.1 (6.4)	27.3 (6.5)
≥30.0	739 (35.0)	240 (53.6)	302 (35.0)	197 (24.7)
Smoking				
Never	1303 (61.8)	273 (60.9)	541 (62.7)	489 (61.2)
Former	435 (20.6)	83 (18.5)	191 (22.1)	161 (20.2)
Current	356 (16.9)	90 (20.1)	124 (14.4)	142 (17.8)
Not reported	16 (0.8)	2 (0.4)	7 (0.8)	7 (0.9)
Alcohol intake, median (IQR), mL/d	2.4 (0-14.5)	0.0 (0-9.5)	2.4 (0-14.3)	5.1 (0-17.4)
Self-rated health, poor/fair	195 (9.2)	77 (17.2)	66 (7.6)	52 (6.5)
Center				
Birmingham, Alabama	399 (18.9)	103 (23.0)	169 (19.6)	127 (15.9)
Chicago, Illinois	504 (23.9)	100 (22.3)	205 (23.8)	199 (24.9)
Minneapolis, Minnesota	538 (25.5)	116 (25.9)	208 (24.1)	214 (26.8)
Oakland, California	669 (31.7)	129 (28.8)	281 (32.6)	259 (32.4)
Systolic blood pressure, mean (SD), mm Hg	115 (14)	116 (15)	115 (14)	114 (13)
Hypertension medication use	324 (15.4)	96 (21.4)	131 (15.2)	97 (12.1)
Hypertension (stage 2)^a^	413 (19.6)	121 (27.0)	167 (19.4)	125 (15.6)
Hyperlipidemia^b^	1189 (56.4)	267 (59.6)	471 (54.6)	451 (56.4)
Diabetes^c^	166 (7.9)	71 (15.8)	60 (7.0)	35 (4.4)
Cardiovascular disease^d^	25 (1.2)	9 (2.0)	10 (1.2)	6 (0.8)
Step volume, median (IQR), steps/d	9146 (7307-11 162)	5837 (5166-6392)	8502 (7822-9278)	11 815 (10 826-13 588)
Stepping intensity, median (IQR)				
Peak 30-min steps/min	75.7 (65.3-87.9)	59.5 (52.1-67.1)	73.88 (66.3-82.4)	88.8 (77.8-99.0)
Time spent at ≥100 steps/min, min/d	6.7 (2.9-14.9)	2.5 (1.1-4.9)	6.5 (3.2-12.0)	14.0 (5.7-24.5)
Moderate to vigorous physical activity, min/d	28.1 (16.3-44.2)	14.0 (9.3-20.6)	25.1 (16.7-36.3)	48.7 (31.1-61.7)
Adherence, mean (SD)				
Wear, min/d	862.4 (84.6)	821.4 (81.0)	872.6 (78.8)	885.2 (84.2)
Wear days	7.0 (1.4)	6.6 (1.6)	7.1 (1.3)	7.1 (1.3)

Abbreviations: BMI, body mass index (calculated as weight in kilograms divided by height in meters squared); IQR, interquartile range.

^a^ Stage 2 hypertension was defined as systolic blood pressure of 140 mm Hg or greater, diastolic blood pressure of 90 mm Hg or greater, and/or using hypertension medication.

^b^ Hyperlipidemia was defined as total cholesterol level of 249 mg/dL or greater (to convert to millimoles per liter, multiply by 0.0259) and/or using cholesterol lowering medications.

^c^ Diabetes was defined as fasting plasma glucose level of 126 mg/dL or greater (to convert to millimoles per liter, multiply by 0.0555), oral glucose tolerance test 200 mg/dL or greater, hemoglobin A_1c_ of 6.5% or greater (to convert to portion of total hemoglobin, multiply by 0.01), and/or using diabetes medication.

^d^ Cardiovascular disease includes adjudicated events prior to the year 20 visit.

**Figure 1.**
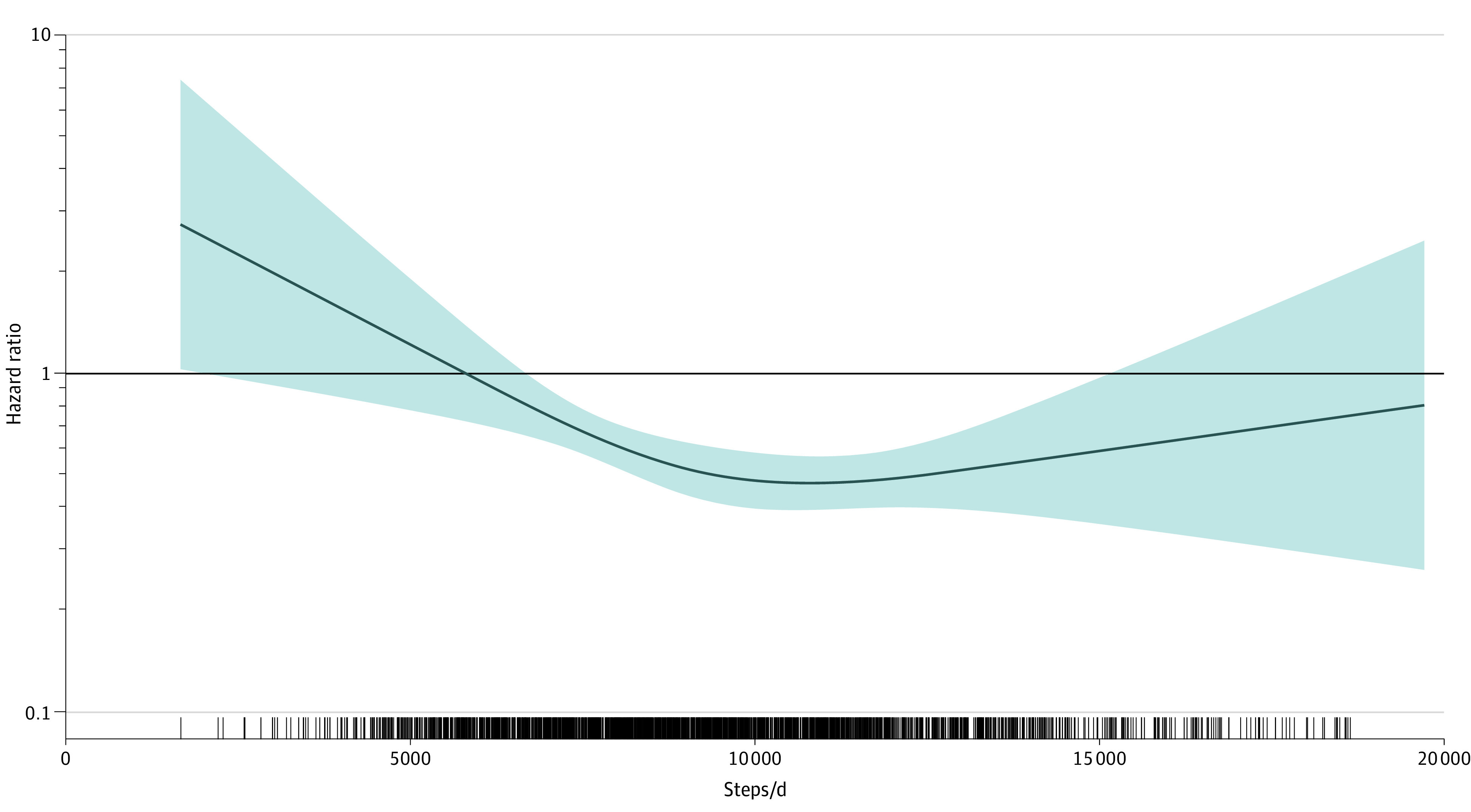
Dose-Response Association of Steps per Day With All-Cause Mortality Restricted cubic splines of hazard ratios of steps/d with all-cause mortality. Knots set at 10th, 50th, and 90th percentile of steps per day. Reference set at 5800 steps/d (the approximate median steps per day of low step group). The model is adjusted for age, accelerometer wear time, race, sex, education, study center, body mass index, smoking, alcohol, systolic blood pressure, hypertension medication use, diabetes, hyperlipidemia, history of cardiovascular disease, and self-rated health. Shading indicates 95% CI.

In our primary results, compared with participants in the low step group and after accounting for all covariates, there was significantly lower risk of mortality for participants in moderate (HR, 0.28 [95% CI, 0.15-0.54]]; risk difference [RD], 53 [95% CI, 27-78] events per 1000 people) and high (HR, 0.45 [95% CI, 0.25-0.81]; RD, 41 [95% CI, 15-68] events per 1000 people) step groups. (Table 2; eTable 3 in the Supplement). Similar findings were observed in sensitivity analyses excluding participants who died within the first 2 years (eTable 4 in the Supplement), using time-varying covariates in extended proportional hazards models (eTable 5 in the Supplement), and adjusting for diet (eTable 6 in the Supplement). Spline analyses found a decline across the distribution of steps up to approximately 10 000 steps/d (Figure 1) and was similar for all demographic groups (eFigure 2 in the Supplement).

**Table 2.** Association of Step Volume With All-Cause Mortality

Step group	Participants, No.	Cases/person-years	Risk difference/1000 people (95% CI)	Hazard ratio (95% CI)		
				Model 1^a^	Model 2^b^	Model 3^c^
<7000 steps/d	448	32/4796	[Reference]	1 [Reference]	1 [Reference]	1 [Reference]
7000-9999 steps/d	863	16/9384	53 (27-78)	0.24 (0.13-0.46)	0.27 (0.14-0.50)	0.28 (0.15-0.54)
≥10 000 steps/d	799	24/8665	41 (15-68)	0.39 (0.22-0.70)	0.40 (0.23-0.72)	0.45 (0.25-0.81)
*P* value for trend	NA	NA	NA	<.01	<.01	.01

Abbreviation: NA, not applicable.

^a^ Adjusted for age, accelerometer wear time, race, and sex.

^b^ Adjusted for variables included in model 1 + education, study center, body mass index, smoking, and alcohol.

^c^ Adjusted for variables included in model 2 + systolic blood pressure, hypertension medication use, diabetes, hyperlipidemia, history of cardiovascular disease, and self-rated health.

**Figure 2.**
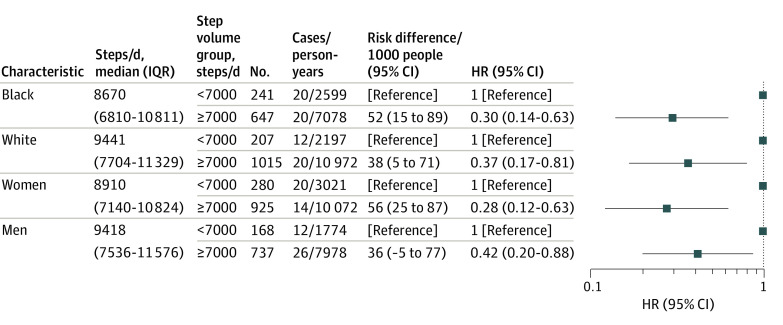
Associations of Steps per Day With All-Cause Mortality by Race and by Sex Hazard ratio (HR) and 95% CIs are adjusted for age, accelerometer wear time, race (for sex models), sex (for race models), education, center, body mass index, smoking, alcohol, systolic blood pressure, history of cardiovascular disease, and self-rated health. *P* < .001 for difference in steps per day between Black vs White participants and women vs men.

Black participants took fewer steps than White participants (median [IQR], 8670 [6810-10 811] steps/d vs 9441 [7704-11 329] steps/d; *P* < .001) (Figure 2). Women took fewer steps than men (median [IQR], 8910 [7140-10 842] steps/d vs 9418 [7536-11 576] steps/d; *P* < .001) (Figure 2). In race-specific analysis, compared with participants in the low step group, high step rates were associated with reduced risk among Black participants (HR, 0.30 [95% CI, 0.14- 0.63]) and White participants (HR, 0.37 [95% CI, 0.17- 0.81]) (Figure 2). In sex-specific analysis, compared with participants in the low step group, high step rates were associated with reduced risk for women (HR, 0.28 [95% CI, 0.12- 0.63]) and men (HR, 0.42 [95% CI, 0.20-0.88]) (Figure 2). There was no association between step intensity and mortality, regardless of adjustment for step volume (Table 3).

**Table 3.** Association of All-Cause Mortality by Daily Stepping Intensity Tertiles

Stepping intensity group	Mean (SD)	Participants, No.	Events	HR (95% CI)		Residual method adjusted for step volume		
				Model 1^a^	Model 3^b^	No.	Events	Model 4, HR (95% CI)^c^
Peak 30 min steps/min, tertile								
1	59.3 (7.7)^d^	703	33	1 [Reference]	1 [Reference]	703	33	1 [Reference]
2	75.8 (4.2)^d^	704	14	0.46 (0.25-0.87)	0.58 (0.30-1.10)	704	19	0.68 (0.38-1.21)
3	96.6 (12.1)^d^	703	25	0.85 (0.49-1.45)	1.17 (0.66-2.09)	703	20	0.98 (0.54-1.77)
*P* value for trend	NA	NA	NA	.45	.73	NA	NA	.78
Time spent at ≥100 steps/min, tertile								
1	1.9 (1.1)^e^	697	33	1 [Reference]	1 [Reference]	703	22	1 [Reference]
2	7.0 (2.2)^e^	709	15	0.49 (0.26-0.90)	0.61 (0.33-1.14)	704	28	1.33 (0.72-2.47)
3	22.9 (11.5)^e^	704	24	0.78 (0.45-1.36)	1.09 (0.61-1.95)	703	22	1.38 (0.73-2.61)
*P* value for trend	NA	NA	NA	.33	.89	NA	NA	.33

Abbreviations: HR, hazard ratio; NA, not applicable.

^a^ Adjusted for age, sex, race, accelerometer wear time.

^b^ Adjusted for variables included in Model 1 + education, center, body mass index, smoking, alcohol, systolic blood pressure, hypertension medication use, diabetes, hyperlipidemia, history of cardiovascular disease, and self-rated health.

^c^ Adjusted for variables included in model 3 + step volume using the residual method for intensity.

^d^ Presented as steps per minute.

^e^ Presented as minutes per day.

## Discussion

In this cohort study of Black and White middle-aged women and men, higher daily steps were associated with a lower risk of all-cause mortality. Adults taking at least 7000 steps/d, compared with those taking fewer than 7000 steps/d, had approximately 50% to 70% lower risk of mortality. Taking more than 10 000 steps/d was not associated with further reduction in mortality risk. This work extends previous research on the association between steps and mortality in a prospective study of middle-aged Black and White adults.

Our findings are consistent with a nationally representative prospective cohort study of men and women from NHANES, using the same ActiGraph 7164 device.^4^ Similar to our findings, the NHANES study of 6355 men and women (mean age, 57 years) found that higher step volumes of 8000 to 12 000 steps/d were associated with approximately 50% to 65% reduction in risk of mortality compared with the 4000 step/d group.^4^ These findings were similar across racial and ethnic groups and for women and men. In the NHANES study,^4^ women, men, non-Hispanic Black, and non-Hispanic White participants taking 8000 steps/d (vs 4000 steps/d) were at 52% to 55% reduced risk of mortality.^4^ We found similar dose-response curves to those in the NHANES study, with risk reductions leveling off at approximately 10 000 steps/d. Conversely, the Women’s Health Study^14^ reported a leveling at 7500 steps/d, which could be owing to the older age of participants (mean age, 72 years). Older adults may require a lower volume of activity to gain similar improvements in fitness and functional status and achieve health benefits.

Our findings support those of previous studies, suggesting that increasing steps per day among the least active portion of the population may provide mortality benefit.^3,4,5,12^ For example, the Norwegian Physical Activity Surveillance Study^5^ showed the largest risk reduction between the lowest quartile and the second-lowest quartile of 48% reduced risk, with risk reductions up to 57% in the highest quartile. In this study, we did not investigate the potential mortality benefit of taking fewer than 7000 steps/d because of the sample size and our younger, more active population.^16^ Studies with larger sample sizes than CARDIA, such as the study of NHANES participants^4^ and older adult women in the Women’s Health Study,^12^ were able to examine participants in 1000- to 2000-increment step groups, starting from fewer than 2000 steps/d. These 2 studies found individuals with as few as approximately 4000 steps/d had approximately 30% lower risk of mortality compared with individuals with approximately 2000 steps/d.

Studies of steps and mortality often lack diversity.^3^ The CARDIA study offers a unique opportunity to study the potential interaction of race and sex with the association of steps and mortality. We did not observe statistically significant interactions by sex or race. We did not observe any significant differences in mortality risk when comparing Black (HR, 0.30) and White (HR, 0.37) participants or comparing women (HR, 0.28) and men (HR, 0.42) according to the 7000 steps/d or more groups vs the fewer than 7000 steps/d referent group. The smaller number of deaths may have contributed to the lack of significant results.

Consistent with studies using wearable monitors to measure steps,^4,12^ stepping intensity in this study was not associated with mortality. In contrast, faster self-reported walking speeds have been associated with lower risks of mortality.^10,11^ In our study, participants with higher stepping intensity tended to take more steps, making it difficult to ascertain the independent association of volume and intensity with mortality. Cross-sectional evidence suggests that stepping intensity may be beneficial for other outcomes, such as cardiometabolic risk factors, which have implications for mortality.^33,34,35^ Prospective studies may be needed to examine the associations of step volume and intensity with cardiometabolic outcomes and other clinical end points.

The findings of this study may have important clinical implications. Wearable patient monitoring systems are emerging as personalized medicine tools for the prevention and management of chronic conditions.^36^ Steps estimated from these devices could be a simple metric to track and promote physical activity. Encouraging walking to achieve step goals is a well-tolerated form of activity for most people. The popularity of consumer wearables has increased over the past decade; for example, users of wearable Fitbit activity trackers have increased from approximately a half million people in 2012 to 29.5 million people in 2019.^37^ It is important to provide evidence-based recommendations for the number and intensity of steps associated with mortality and other health benefits.

This study has several strengths. With a mean follow-up of 10.8 years, this is a relatively long follow-up duration for studies on associations of steps with mortality.^3^ The CARDIA study used a device validated against criterion standards to measure steps and a wealth of covariate data. Our results demonstrated associations of steps with mortality across race and sex subgroups. With the younger cohort than in previous studies, we added new evidence focusing on deaths occurring earlier than the mean life expectancy, or premature mortality.^3^

### Limitations

This study has several limitations. The observational design limits conclusions regarding the causal pathway of the association of steps with mortality. The lowest step group had the highest rates of cardiovascular disease, hypertension, and diabetes. Although our analyses attempted to control for these and other health status factors, there remains potential for residual confounding and reverse causality. The low event rate (72 deaths [3.4% of sample]) is expected, given the mean (SD) age at follow-up was 56 (3.6) years (range, 41-66 years). This precluded detailed examination by race-sex groups (eg, Black Women) and cause-specific mortality. As demonstrated with the splines, the 95% CIs widened between 12 000 to 15 000 steps/d, limiting inferences on higher volumes of steps per day with mortality. Selection bias may have resulted if participants died or dropped out prior to the year 20 visit or did not wear the accelerometer during year 20. The algorithm used to analyze the accelerometer data may not adequately capture bouts less than 1 minute, and short walking bouts are common in daily life.^38^ The intensity metrics were developed in controlled settings of treadmill walking at constant speeds. This may not adequately represent free-living stepping intensity and sporadic patterns of walking behaviors.

The ActiGraph 7164 is a wearable activity monitor validated to measure steps in free living conditions compared with the criterion standard device, StepWatch.^17^ Step counts from various research and commercial monitors are highly correlated (>0.80) but not identical to those from other wearable activity monitors. The ActiGraph 7164 device can estimate 15% to 25% more steps compared with other common devices.^17,39^ Therefore, the specific step count values provided in this study may not be precisely applicable to every wearable activity monitor.

## Conclusions

This cohort study among Black and White men and women found that taking at least 7000 steps/d during middle adulthood was associated with a lower risk of mortality. There was no association of step intensity with mortality. Improving physical activity levels in the least active segment of the population by encouraging increasing steps/d may be associated with lower mortality risk.

## Notes

Supplement. **eFigure 1.** Flowchart of Sample Selection Among CARDIA Study Participants **eFigure 2.** Dose-Response Association of Steps per Day with Premature All-Cause Mortality by Race and by Sex **eTable 1.** Participant Characteristics at Year 20 Among Individuals Included vs Excluded Based on Accelerometer Wear **eTable 2.** Summary of Cause of Death for Total Sample and by Step Groups and Race and Sex Groups **eTable 3.** Hazard Ratios for the Association of All-Cause Mortality With Details of Parameters Included in Model **eTable 4.** Dose-Response Association of Steps per Day With Premature All-Cause Mortality Excluding Individuals Who Died in First 2 Years **eTable 5.** Extended Cox Regression Model Using Time Varying Covariates for the Dose-Response Association of Steps per Day With Premature All-Cause Mortality **eTable 6.** Dose-Response Association of Steps per Day With Premature All-Cause Mortality Among Subsample Sample With Diet Data Available

## References

[1] 2018 Physical Activity Guidelines Advisory Committee. 2018 Physical Activity Guidelines Advisory Committee Scientific Report. US Department of Health and Human Services, 2018.

[2] Bassett DR Jr, Toth LP, LaMunion SR, Crouter SE. Step counting: a review of measurement considerations and health-related applications. Sports Med. 2017;47(7):1303-1315. doi:10.1007/s40279-016-0663-1 28005190 PMC5488109

[3] Hall KS, Hyde ET, Bassett DR, . Systematic review of the prospective association of daily step counts with risk of mortality, cardiovascular disease, and dysglycemia. Int J Behav Nutr Phys Act. 2020;17(1):78. doi:10.1186/s12966-020-00978-9 32563261 PMC7305604

[4] Saint-Maurice PF, Troiano RP, Bassett DR Jr, . Association of daily step count and step intensity with mortality among US adults. JAMA. 2020;323(12):1151-1160. doi:10.1001/jama.2020.1382 32207799 PMC7093766

[5] Hansen BH, Dalene KE, Ekelund U, . Step by step: association of device-measured daily steps with all-cause mortality—a prospective cohort study. Scand J Med Sci Sports. 2020;30(9):1705-1711. doi:10.1111/sms.13726 32427398 PMC7496562

[6] Kraus WE, Janz KF, Powell KE, ; 2018 Physical Activity Guidelines Advisory Committee. Daily step counts for measuring physical activity exposure and its relation to health. Med Sci Sports Exerc. 2019;51(6):1206-1212. doi:10.1249/MSS.0000000000001932 31095077 PMC6527133

[7] Arias E, Xu J. United States life tables, 2018. Natl Vital Stat Rep. 2020;69(12):1-45. 33270553

[8] Piercy KL, Troiano RP, Ballard RM, . The physical activity guidelines for Americans. JAMA. 2018;320(19):2020-2028. doi:10.1001/jama.2018.14854 30418471 PMC9582631

[9] Wang Y, Nie J, Ferrari G, Rey-Lopez JP, Rezende LFM. Association of physical activity intensity with mortality: a national cohort study of 403 681 US adults. JAMA Intern Med. 2021;181(2):203-211. doi:10.1001/jamainternmed.2020.6331 33226432 PMC7684516

[10] Elbaz A, Sabia S, Brunner E, . Association of walking speed in late midlife with mortality: results from the Whitehall II cohort study. Age (Dordr). 2013;35(3):943-952. doi:10.1007/s11357-012-9387-9 22361996 PMC3636402

[11] Yates T, Zaccardi F, Dhalwani NN, . Association of walking pace and handgrip strength with all-cause, cardiovascular, and cancer mortality: a UK Biobank observational study. Eur Heart J. 2017;38(43):3232-3240. doi:10.1093/eurheartj/ehx449 29020281 PMC5837337

[12] Lee IM, Shiroma EJ, Kamada M, Bassett DR, Matthews CE, Buring JE. Association of step volume and intensity with all-cause mortality in older women. JAMA Intern Med. 2019;179(8):1105-1112. doi:10.1001/jamainternmed.2019.0899 31141585 PMC6547157

[13] Stensvold D, Viken H, Steinshamn SL, . Effect of exercise training for five years on all cause mortality in older adults—the Generation 100 study: randomised controlled trial. BMJ. 2020;371:m3485. doi:10.1136/bmj.m3485 33028588 PMC7539760

[14] Chodzko-Zajko WJ, Proctor DN, Fiatarone Singh MA, ; American College of Sports Medicine. American College of Sports Medicine position stand: exercise and physical activity for older adults. Med Sci Sports Exerc. 2009;41(7):1510-1530. doi:10.1249/MSS.0b013e3181a0c95c 19516148

[15] Friedman GD, Cutter GR, Donahue RP, . CARDIA: study design, recruitment, and some characteristics of the examined subjects. J Clin Epidemiol. 1988;41(11):1105-1116. doi:10.1016/0895-4356(88)90080-7 3204420

[16] Pettee Gabriel K, Sidney S, Jacobs DR Jr, . Ten-year changes in accelerometer-based physical activity and sedentary time during midlife: the CARDIA study. Am J Epidemiol. 2018;187(10):2145-2150. doi:10.1093/aje/kwy117 29893772 PMC6166210

[17] Feito Y, Bassett DR, Thompson DL. Evaluation of activity monitors in controlled and free-living environments. Med Sci Sports Exerc. 2012;44(4):733-741. doi:10.1249/MSS.0b013e3182351913 21904249

[18] Troiano RP, Berrigan D, Dodd KW, Mâsse LC, Tilert T, McDowell M. Physical activity in the United States measured by accelerometer. Med Sci Sports Exerc. 2008;40(1):181-188. doi:10.1249/mss.0b013e31815a51b3 18091006

[19] National Cancer Institute. SAS programs for analyzing NHANES 2003-2004 accelerometer data. Accessed August 3, 2021. https://epi.grants.cancer.gov/nhanes-pam/

[20] Jefferis BJ, Parsons TJ, Sartini C, . Objectively measured physical activity, sedentary behaviour and all-cause mortality in older men: does volume of activity matter more than pattern of accumulation? Br J Sports Med. 2019;53(16):1013-1020. doi:10.1136/bjsports-2017-098733 29440040 PMC6691867

[21] Tudor-Locke C, Craig CL, Brown WJ, . How many steps/day are enough: for adults. Int J Behav Nutr Phys Act. 2011;8:79. doi:10.1186/1479-5868-8-79 21798015 PMC3197470

[22] Tudor-Locke C, Camhi SM, Troiano RP. A catalog of rules, variables, and definitions applied to accelerometer data in the National Health and Nutrition Examination Survey, 2003-2006. Prev Chronic Dis. 2012;9:E113. doi:10.5888/pcd9.110332 22698174 PMC3457743

[23] Pettee Gabriel K, Whitaker KM, Duprez D, . Clinical importance of non-participation in a maximal graded exercise test on risk of non-fatal and fatal cardiovascular events and all-cause mortality: CARDIA study. Prev Med. 2018;106:137-144. doi:10.1016/j.ypmed.2017.10.025 29080827 PMC6400469

[24] McDonald A, Van Horn L, Slattery M, . The CARDIA dietary history: development, implementation, and evaluation. J Am Diet Assoc. 1991;91(9):1104-1112. 1918764

[25] Carnethon MR, Loria CM, Hill JO, Sidney S, Savage PJ, Liu K; Coronary Artery Risk Development in Young Adults study. Risk factors for the metabolic syndrome: the Coronary Artery Risk Development in Young Adults (CARDIA) study, 1985-2001. Diabetes Care. 2004;27(11):2707-2715. doi:10.2337/diacare.27.11.2707 15505009

[26] Folsom AR, Jacobs DR Jr, Wagenknecht LE, . Increase in fasting insulin and glucose over seven years with increasing weight and inactivity of young adults: the CARDIA study—Coronary Artery Risk Development in Young Adults. Am J Epidemiol. 1996;144(3):235-246. doi:10.1093/oxfordjournals.aje.a008918 8686692

[27] Whelton PK, Carey RM, Aronow WS, . 2017 ACC/AHA/AAPA/ABC/ACPM/AGS/APhA/ASH/ASPC/NMA/PCNA guideline for the prevention, detection, evaluation, and management of high blood pressure in adults: executive summary: a report of the American College of Cardiology/American Heart Association Task Force on Clinical Practice Guidelines. Circulation. 2018;138(17):e426-e483. doi:10.1161/CIR.0000000000000597 30354655

[28] Grundy SM, Stone NJ, Bailey AL, . 2018 AHA/ACC/AACVPR/AAPA/ABC/ACPM/ADA/AGS/APhA/ASPC/NLA/PCNA guideline on the management of blood cholesterol: a report of the American College of Cardiology/American Heart Association Task Force on Clinical Practice Guidelines. Circulation. 2019;139(25):e1082-e1143. doi:10.1161/CIR.0000000000000625 30586774 PMC7403606

[29] American Diabetes Association. 2. Classification and diagnosis of diabetes: *Standards of Medical Care in Diabetes—2020.* Diabetes Care. 2020;43(suppl 1):S14-S31. doi:10.2337/dc20-S002 31862745

[30] Harrell FJ. rms: Regression modeling strategies. Accessed August 3, 2021. https://cran.r-project.org/web/packages/rms/index.html

[31] Desquilbet L, Mariotti F. Dose-response analyses using restricted cubic spline functions in public health research. Stat Med. 2010;29(9):1037-1057. doi:10.1002/sim.3841 20087875

[32] Willett WC, Howe GR, Kushi LH. Adjustment for total energy intake in epidemiologic studies. Am J Clin Nutr. 1997;65(4)(suppl):1220S-1228S. doi:10.1093/ajcn/65.4.1220S 9094926

[33] Adams B, Fidler K, Demoes N, . Cardiometabolic thresholds for peak 30-min cadence and steps/day. PLoS One. 2019;14(8):e0219933. doi:10.1371/journal.pone.0219933 31374078 PMC6677301

[34] Tudor-Locke C, Schuna JM Jr, Han HO, . Step-based physical activity metrics and cardiometabolic risk: NHANES 2005-2006. Med Sci Sports Exerc. 2017;49(2):283-291. doi:10.1249/MSS.0000000000001100 27669450 PMC5412514

[35] Sumner J, Uijtdewilligen L, Yee ACH, . Volume and intensity of stepping activity and cardiometabolic risk factors in a multi-ethnic Asian population. Int J Environ Res Public Health. 2020;17(3):E863. doi:10.3390/ijerph17030863 32019086 PMC7037023

[36] Baig MM, GholamHosseini H, Moqeem AA, Mirza F, Lindén M. A systematic review of wearable patient monitoring systems—current challenges and opportunities for clinical adoption. J Med Syst. 2017;41(7):115. doi:10.1007/s10916-017-0760-1 28631139

[37] Statista. Number of active users of Fitbit from 2012 to 2019. Accessed October 7, 2020. https://www.statista.com/statistics/472600/fitbit-active-users/

[38] Dall PM, McCrorie PR, Granat MH, Stansfield BW. Step accumulation per minute epoch is not the same as cadence for free-living adults. Med Sci Sports Exerc. 2013;45(10):1995-2001. doi:10.1249/MSS.0b013e3182955780 23568091

[39] Hickey A, John D, Sasaki JE, Mavilia M, Freedson P. Validity of activity monitor step detection is related to movement patterns. J Phys Act Health. 2016;13(2):145-153. doi:10.1123/jpah.2015-0203 26107045

