# Supplementary material for: Steps per Day and All-Cause Mortality in Middle-aged Adults in the Coronary Artery Risk Development in Young Adults Study
Source: JAMA Netw Open. 2021 Sep 3;4(9):e2124516. doi: 10.1001/jamanetworkopen.2021.24516 (PMC8417757; doi:10.1001/jamanetworkopen.2021.24516)
Supplement: Supplement. — eFigure 1. Flowchart of Sample Selection Among CARDIA Study Participants eFigure 2. Dose-Response Association of Steps per Day with Premature All-Cause Mortality by Race and by Sex eTable 1. Participant Characteristics at Year 20 Among Individuals Included vs Excluded Based on Accelerometer Wear eTable 2. Summary of Cause of Death for Total Sample and by Step Groups and Race and Sex Groups eTable 3. Hazard Ratios for the Association of All-Cause Mortality With Details of Parameters Included in Model eTable 4. Dose-Response Association of Steps per Day With Premature All-Cause Mortality Excluding Individuals Who Died in First 2 Years eTable 5. Extended Cox Regression Model Using Time Varying Covariates for the Dose-Response Association of Steps per Day With Premature All-Cause Mortality eTable 6. Dose-Response Association of Steps per Day With Premature All-Cause Mortality Among Subsample Sample With Diet Data Available [file jamanetwopen-e2124516-s001.pdf]

Supplemental Online Content

Paluch AE, Gabriel KP, Fulton JE, et al. Steps per day and all-cause mortality in middle-aged adults in the Coronary Artery Risk Development in Young Adults Study. *JAMA Netw Open*. 2021;4(9):e2124516. doi:10.1001/jamanetworkopen.2021.24516

eFigure 1. Flowchart of Sample Selection Among CARDIA Study Participants

eFigure 2. Dose-Response Association of Steps per Day with Premature All-Cause Mortality by Race and by Sex

eTable 1. Participant Characteristics at Year 20 Among Individuals Included vs Excluded Based on Accelerometer Wear

eTable 2. Summary of Cause of Death for Total Sample and by Step Groups and Race and Sex Groups

eTable 3. Hazard Ratios for the Association of All-Cause Mortality With Details of Parameters Included in Model

eTable 4. Dose-Response Association of Steps per Day With Premature All-Cause Mortality Excluding Individuals Who Died in First 2 Years

eTable 5. Extended Cox Regression Model Using Time Varying Covariates for the Dose-Response Association of Steps per Day Wwith Premature All-Cause Mortality

eTable 6. Dose-Response Association of Steps per Day With Premature All-Cause Mortality Among Subsample Sample With Diet Data Available

This supplemental material has been provided by the authors to give readers additional information about their work.

© 2021 Paluch AE et al. *JAMA Network Open.*

eFigure 1. Flowchart of Sample Selection Among CARDIA Study Participants

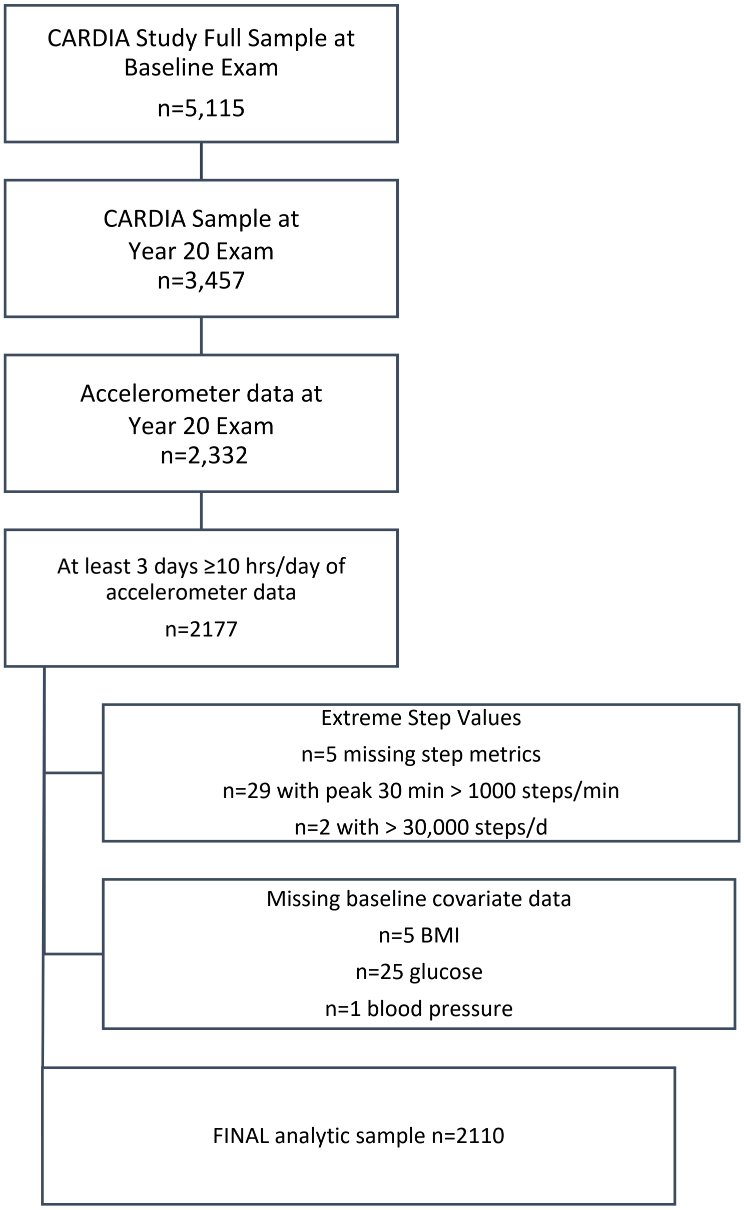

© 2021 Paluch AE et al. *JAMA Network Open.*

eFigures 2. Dose Response Association of Steps per Day with Premature All-Cause Mortality by Race and by Sex

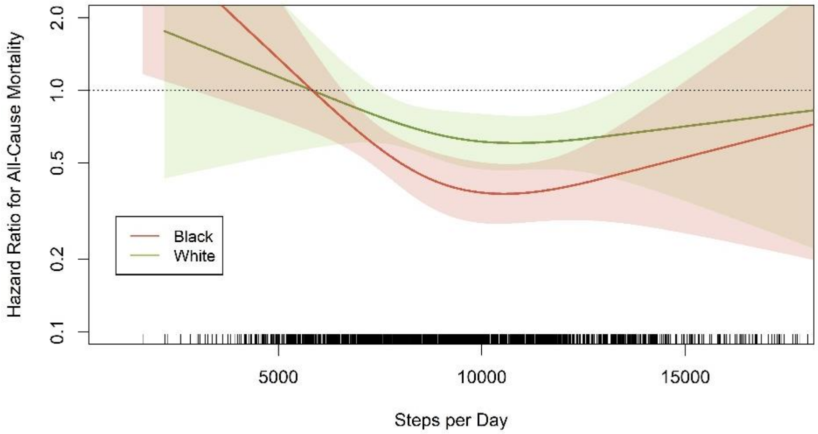

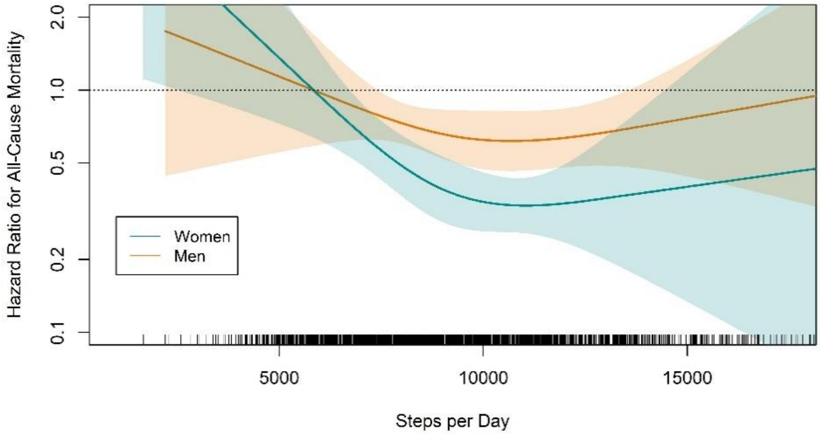

Restricted Cubic Splines of Hazard Ratios of steps/d with all-cause mortality. Knots set at 10th, 50th, and 90th percentile of steps/d. Reference set at 5800 steps/d (~median steps/d of low step group). Model is adjusted for age, accelerometer wear time, race (for sex models), sex (for race models), education, study center, BMI, smoking, alcohol, Systolic BP, Hypertension medication, Diabetes, Hyperlipidemia, History of CVD, Self-Rated Health. The y-axis is a log scale. Data are shown as hazard ratios and 95% confidence intervals.

© 2021 Paluch AE et al. *JAMA Network Open.*

eTable 1. Participant Characteristics at Year 20 Among Individuals Included vs Excluded Based on Accelerometer Wear

	**Included (n=2110)**	**Excluded (n=765)**
**Baseline age, years**	45.2 (3.6)	45.2 (3.6)
**Women, n (%)**	1205 (57.1)	430 (56.2)
**Black, n (%)***	888 (42.1)	401 (52.4)
**Education, years***	15.6 (2.6)	15.4 (2.7)
**Current Smoker, n (%)***	356 (16.9)	160 (20.9)
**Alcohol consumption, mL/day, median [IQR]**	2.4 [0, 14.5]	2.4 [0, 12.2]
**Self-reported Physical Activity, Exercise Units***	355 (274)	310 (268)
**BMI, kg/m^2**	29.0 (7.0)	29.3 (6.7)
**Systolic Blood Pressure, mmHg**	115 (14)	115 (15)
**Self-reported Health, poor/fair, n (%)***	195 (9.2)	91 (11.9)
**Hyperlipidemia, n (%)**	1189 (56.4)	461 (60.3)
**Diabetes, n (%)**	166 (7.9)	60 (7.8)
**History of CVD, n (%)**	25 (1.2)	14 (1.8)
**Deaths, n (%)**	72 (3.4)	35 (4.5)

*** indicates significant differences p value <0.05**
Continuous variables reported as means (s.d.) unless noted
Excluded participants are considered those who did not wear the accelerometer or had less than 3 days of ≥10 hrs/d of accelerometer data recorded at the year 20 examination.

© 2021 Paluch AE et al. *JAMA Network Open.*

eTable 2. Summary of Cause of Death for Total Sample and by Step Groups and Race and Sex Groups

	**Total Sample**	**By Steps/d Group**			**By Race-Sex Groups**			
		**Low <7,000**	**Mod 7,000-9,999**	**High ≥10,000**	**Black Women**	**White Women**	**Black Men**	**White Men**
N	2110	448	863	799	556	636	313	554
Total Deaths	72	32	16	24	20	13	19	19
Cause of Death								
Cardiovascular Disease	17	7	5	5	4	1	5	7
Cancer	18	9	2	7	4	8	3	3
Type 2 Diabetes	1	1	0	0	1	0	0	0
Kidney Disease	1	1	0	0	1	0	0	0
AIDS	3	1	1	1	0	0	1	2
Homicide	2	1	0	1	1	0	1	0
Liver Disease	4	2	1	1	0	1	0	2
Asthmas	2	1	1	0	1	0	1	0
Other Lung Disease	3	1	1	1	1	1	1	0
Suicide	2	0	0	2	0	2	0	0
Unintentional Injury	5	2	3	0	0	0	1	4
Sepsis	5	3	0	2	1	0	3	1
Other	1	0	0	1	0	0	1	0
Unknown	8	3	2	3	6	0	2	0

© 2021 Paluch AE et al. *JAMA Network Open.*

eTable 3. Hazard Ratios for the Association of All-Cause Mortality With Details of Parameters Included in Model

	Model 2				Model 3			
Parameter	**Hazard Ratio**	**95% Confidence Interval**		**P-value**	**Hazard Ratio**	**95% Confidence Interval**		**P-value**
Step group moderate (vs. low (ref))	0.270	0.144	0.508	0.001	0.285	0.150	0.541	0.008
Step group high (vs. low (ref))	0.402	0.225	0.720	0.002	0.446	0.246	0.809	0.001
Age	1.146	1.066	1.232	0.001	1.136	1.056	1.224	0.001
Sex (Female vs Male(ref))	0.625	0.378	1.032	0.066	0.594	0.353	1.000	0.050
Race (White vs Black (ref))	0.788	0.473	1.313	0.360	0.781	0.455	1.338	0.367
Education	0.894	0.809	0.987	0.026	0.910	0.825	1.004	0.060
Field Center (Birmingham vs Oakland (ref))	2.409	1.219	4.764	0.011	2.436	1.230	4.826	0.011
Field Center (Chicago vs Oakland (ref)	1.193	0.571	2.492	0.638	1.203	0.572	2.527	0.626
Field Center (Minneapolis vs Oakland (ref))	1.665	0.837	3.312	0.146	1.557	0.775	3.126	0.213
Accelerometer wear time (min/d)	1.001	0.998	1.004	0.434	1.001	0.998	1.003	0.599
BMI (kg/m^2)	1.015	0.986	1.045	0.302	1.011	0.980	1.042	0.492
Smoking Status (former vs never (ref))	0.947	0.487	1.842	0.872	0.950	0.487	1.855	0.881
Smoking Status (current vs never (ref))	1.570	0.878	2.807	0.128	1.432	0.785	2.614	0.241
Alcohol intake (none vs >12 ml/d (ref))	0.856	0.478	1.532	0.600	0.832	0.464	1.491	0.535
Alcohol intake (<12 ml/d vs >12 ml/d (ref))	0.679	0.346	1.331	0.259	0.649	0.330	1.275	0.209
Blood pressure medication (no vs yes (ref))					1.249	0.650	2.402	0.504
Diabetes (no vs. yes (ref))					0.989	0.455	2.150	0.978
Hyperlipidemia (no vs. yes (ref))					1.370	0.822	2.284	0.226
History of CVD (no vs. year (ref))					0.304	0.110	0.839	0.021
Systolic Blood Pressure (mmHg)					1.004	0.989	1.019	0.573
Self-Rated Health (poor/fair vs good/very good/excellent (ref))					1.592	0.831	3.052	0.161
Likelihood Ratio Test[a]								
Comparing to nested model 1				0.001				0.002
Comparing to nested model 2				-				0.261

[a]Likelihood ratio test for nested model comparison. P-values calculated using a chi-squared test for nested vs full model. Model 1adjusted for age, sex, race, accelerometer wear time.

© 2021 Paluch AE et al. *JAMA Network Open.*

eTable 4. Dose-Response Association of Steps per Day With Premature All-Cause Mortality Excluding Individuals Who Died in First 2 Years

	No.	Events	Model 1<br>HR (95% CI)	Model 3<br>HR (95% CI)
**<7,000 steps/d**	447	31	1.00 (ref)	1.00 (ref)
**7,000-9,999 steps/d**	862	15	0.22 (0.12 - 0.42)	0.27 (0.14 - 0.52)
**≥10,000 steps/d**	798	23	0.37 (0.21 - 0.63)	0.42 (0.23 - 0.76)
**p-value for trend**			<0.01	<0.01

Model 1: Adjust models for age, sex, race, accelerometer wear time
Model 3: Model 1 + education, center, BMI, smoking, alcohol intake, systolic blood pressure, Hypertension medication, Diabetes, Hyperlipidemia, CVD, Self-rated Health

© 2021 Paluch AE et al. *JAMA Network Open.*

eTable 5. Extended Cox Regression Model Using Time Varying Covariates for the Dose-Response Association of Steps per Day Wwith Premature All-Cause Mortality

	No.	Events	HR (95% CI) adjusting for time-updated covariates
**<7,000 steps/d**	448	32	1.00 (ref)
**7,000-9,999 steps/d**	863	16	0.28 (0.15 - 0.54)
**≥10,000 steps/d**	779	24	0.44 (0.25 - 0.80)
**p-value for trend**			0.01
**Models adjusted for baseline variables of age, sex, race, accelerometer wear time, education, center, and time-varying variables of BMI, smoking, alcohol, systolic blood pressure, Hypertension medication, Diabetes, Hyperlipidemia, CVD, Self-Rated Health Time-varying variables were measured at year 20, year 25, and year 30 examinations.**			

© 2021 Paluch AE et al. *JAMA Network Open.*

eTable 6. Dose-Response Association of Steps per Day With Premature All-Cause Mortality Among Subsample Sample With Diet Data Available

	n Events/n Total	Healthy Eating Index Included in Model<br>HR (95% CI)	Healthy Eating Index Not Included in Model<br>HR (95% CI)
**<7,000 steps/day**	32/447	1.00 (ref)	1.00 (ref)
**7,000-9,999 Steps/day**	13/862	0.22 (0.11 - 0.43)	0.22 (0.11 - 0.43)
**≥10,000 Steps**	18/798	0.29 (0.15 - 0.54)	0.29 (0.15 - 0.54)
**p-value for trend**		<0.01	<0.01
**Models adjusted for age, sex, race, accelerometer wear time, education, center, BMI, smoking, alcohol (with or without diet variable)**			

© 2021 Paluch AE et al. *JAMA Network Open.*
